# Association between Polycystic Ovary Syndrome and Gut Microbiota

**DOI:** 10.1371/journal.pone.0153196

**Published:** 2016-04-19

**Authors:** Yanjie Guo, Yane Qi, Xuefei Yang, Lihui Zhao, Shu Wen, Yinhui Liu, Li Tang

**Affiliations:** Department of Microecology, School of Basic Medical Science, Dalian Medical University, Dalian, Liaoning, China; Johns Hopkins University School of Medicine, UNITED STATES

## Abstract

Polycystic ovary syndrome (PCOS) is the most frequent endocrinopathy in women of reproductive age. It is difficult to treat PCOS because of its complex etiology and pathogenesis. Here, we characterized the roles of gut microbiota on the pathogenesis and treatments in letrozole (a nonsteroidal aromatase inhibitor) induced PCOS rat model. Changes in estrous cycles, hormonal levels, ovarian morphology and gut microbiota by PCR-DGGE and real-time PCR were determined. The results showed that PCOS rats displayed abnormal estrous cycles with increasing androgen biosynthesis and exhibited multiple large cysts with diminished granulosa layers in ovarian tissues. Meanwhile, the composition of gut microbiota in letrozole-treated rats was different from that in the controls. *Lactobacillus*, *Ruminococcus* and *Clostridium* were lower while *Prevotella* was higher in PCOS rats when compared with control rats. After treating PCOS rats with *Lactobacillus* and fecal microbiota transplantation (FMT) from healthy rats, it was found that the estrous cycles were improved in all 8 rats in FMT group, and in 6 of the 8 rats in *Lactobacillus* transplantation group with decreasing androgen biosynthesis. Their ovarian morphologies normalized. The composition of gut microbiota restored in both FMT and *Lactobacillus* treated groups with increasing of *Lactobacillus* and *Clostridium*, and decreasing of *Prevotella*. These results indicated that dysbiosis of gut microbiota was associated with the pathogenesis of PCOS. Microbiota interventions through FMT and *Lactobacillus* transplantation were beneficial for the treatments of PCOS rats.

## Introduction

Polycystic ovary syndrome (PCOS) is a common endocrine disorder in their reproductive years, affecting 5%-10% of women worldwide [1]. The syndrome is characterized by the presence of at least two of the three classical features: hyperandrogenism, oligo-/anovulation and polycystic ovaries on pelvic ultrasound [2]. Women with PCOS, particularly those with menstrual irregularities may have difficulties conceiving because of anovulation. Besides that, PCOS patients frequently have metabolic disturbances with cardiovascular, type II diabetes, dyslipidemia, visceral obesity and endothelial dysfunction risk factors [3–5]. Therefore, PCOS is not just a cosmetic and fertility problem but also a major health problem that could shorten women’s life expectancy.

The etiology and pathogenesis of PCOS remain unclear and may be multi-factorial, involving genetic, neuroendocrine and metabolic causes [6–9]. Some researchers believe that PCOS may be not an ovarian disease but a metabolic disorder disease [10]. Till now, there is not any unified theory for the pathophysiology of PCOS. What is consistent in PCOS is the production of excess androgens by the ovaries. Further studies are required to investigate the primary pathophysiology mechanisms underlying this syndrome.

Human gut harbors more than 100 trillion microbes referred to as the gut microbiota. However, in the past, the commensal microbiota was largely neglected and mostly inaccessible to investigation. Until recently, researchers realize that these residents in human gut form a symbiotic relationship with the host and provide many benefits to the host. For example, commensal microbes consistently provide a set of services to the host such as modulation of immune system, inhibition of pathogen colonization, liberating nutrients from food [11–13]. Meanwhile, dysbiosis of gut microbiota has been implicated in many disease states, including diabetes, obesity and cardiovascular disease [14–16].

Recently, a novel concept of “microgenderome” related to the potential bidirectional interaction roles between the sex hormones and gut microbiota has emerged [17]. It has been reported that the composition of commensal microbes of male and female animals diverged at the time of puberty, which implied that sex hormone levels exerted specific influences on the composition of the microbiota. Removal of gut microbiota increased the testosterone concentration in female mice but decreased the concentration in male mice. Thus, the commensal gut microbiota also had effects on the production of male sex hormone [18]. It is interesting to explore the role of gut microbiota in PCOS, as the androgen level in PCOS women always elevated. Tremellen and Pearce suggest that dysbiosis of gut microbiota (DOGMA) brought by a high fat-sugar diet in PCOS patients leads to an increase in the intestinal permeability. Lipopolysaccharide produced by gram negative bacteria traverses the “leaky gut” wall into the circulation, leading to a chronic state of low grade inflammation. The activation of immune system interferes with insulin receptor, driving up insulin level, which boosts testosterone production in the ovary leading to PCOS. The DOGMA theory can account for the role of gut microbiota in the pathogenesis of PCOS [19].

On these grounds, we hypothesize that excess androgen biosynthesis in PCOS may result in the dysbiosis of host gut microbiota and modulating of gut microbiota may be beneficial for PCOS treatment. In this study, in order to verify our hypotheses, PCOS rat model was established using letrozole induction. Microbiota interventions through *Lactobacillus* transplantation and fecal microbiota transplantation (FMT) from healthy rats were used for the treatments of PCOS rats. Administration of probiotics such as *Lactobacillus* is an attractive concept in combating various diseases. *L rhamnosus GR-1* attenuated lipopolysaccharide-induced inflammation in pregnant CD-1 mice [20]. *Lactobacillus acidophilus NCFM* maintained insulin sensitivity in a metabolically heterogeneous group [21]. *Lactobacillus rhamnosus GG* and *Lactobacillus casei* DN-114-001 protected epithelial barrier function against *Escherichia coli-*induced redistribution of the tight-junction proteins [22, 23]. Fecal microbiota transplantation (FMT) was introduction of fecal suspension derived from a healthy donor into the gastrointestinal tract of a diseased individual. It has been proposed as a novel therapeutic approach to modulate gut microbiota dysbiosis. Patients with metabolic syndrome increased insulin sensitivity after infusion of microbiota from lean donors [24]. Our previous study showed that FMT promoted the re-establishment of intestinal microbial communities and mucosal barriers in mice with antibiotic-induced dysbiosis [25]. Thus, the available animal and human evidences suggested that *Lactobacillus* transplantation and FMT could serve as new therapies for treating diseases. In the present study, after microbiota interventions through *Lactobacillus* transplantation and FMT, the estrous cycles, sex hormonal levels, ovarian morphology and gut microbiota were examined. We hope that our results will shed a new light on the pathogenesis and treatment for PCOS.

## Materials and Methods

### Animals and animal husbandry

**Fig 1:**
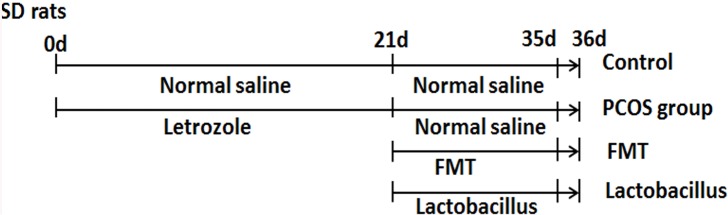
Time lines of the animal experiments. To establish the PCOS rat model, SD rats were treated with letrozole at a concentration of 1 mg/kg once daily for 21 days. After that, PCOS FMT group was treated with 2×10^9^ fecal microbiota once daily, PCOS *Lactobacillus* transplantation group was treated with 2×10^9^ *Lactobacillus* once daily, PCOS group and control group were treated with normal saline for 14 days. On day 21, all rat fecal samples were collected. On day 36, all rat blood samples, ovarian tissue samples and fecal samples were collected.

Six-week-old specific-pathogen free (SPF) level inbred female Sprague-Dawley (SD) rats (mean body weight, 180 g) were purchased from Animal Facility of Dalian Medical University. All animal experiments were approved by the Animal Care Committee of Dalian Medical University, China (SCXK-2013-0003). All animals were feed with commercial diet (51% nitrogen-free extract, 24.9% crude protein, 4.6% crud fat, 6.6% crud ash, 4.1% crud fiber and 8.9% moisture), and tap water *ad libitum*. In this experiment, 32 female SD rats were randomly assigned into 4 groups of 8 rats each, including a control group that received a gavage of normal saline, and three treatment groups (PCOS, PCOS FMT and PCOS *Lactobacillus* transplantation groups) administered a gavage of letrozole (Novartis Pharma Schweiz AG, Switzerland) at a concentration of 1 mg/kg once daily for 21 consecutive days. The establishment of PCOS rat model was similar to that of Kalafi. *et al* [26]. On day 21, all rat fecal samples were collected. Control rat fecal samples and PCOS rat fecal samples were used for DGGE analysis to evaluate the gut microbiota shift in PCOS rats. In order to quantify the differences of microbita for all rats on day 21, real-time PCR analysis was applied. Twenty four hours after the last dose of letrozole (on day 22), PCOS FMT rats were administered a gavage of fecal supernatant with 2×10^9^ fecal microbiota, PCOS *Lactobacillus* transplantation rats were administered a gavage of 2×10^9^ *Lactobacillus*, the control rats and PCOS rats were administered a gavage of normal saline for 14 consecutive days. On day 36, all rat fecal samples were collected for real-time PCR analysis. Then all rats were sacrificed by decapitation. Trunk blood samples and ovarian tissue samples were obtained for the subsequent experiments. The treatments of the animals and sample collections were showed in Fig 1.

### Vaginal smears

Vaginal smears were collected for all of the rats daily at 9:00 am and evaluated microscopically with Giemsa staining for estrous cycle determination. The observation period lasted for 36 consecutive days beginning with the first dose of letrozole till the last transplantation of *Lactobacillus* or fecal microbiota.

### DGGE analysis and sequence analysis

Gut microbiota differences between PCOS rats and control rats were analyzed using DGGE analysis on day 21. The metagenomic DNA was extracted from rat fecal samples with QIAamp DNA stool mini kit (Qiagen, Germany). PCR amplification was conducted using the universal bacterial primers F338+GC clamp and R518 targeting the hyper variable V3 regions of 16S rRNA genes. The PCR products were analyzed using the DCode system (Bio-Rad, USA) according to descriptions of Joossens. *et al*. [27]. DGGE profiles were digitally processed with Quantity One software version 462 in a multistep procedure following the manufacturer’s instructions.

The abundance and relative intensity in the DGGE were analyzed using Phoretix 1D (Phoretix International, UK). To identify some separated and strong bands, bands were excised from the gel and sequenced (Takara, Japan). The sequences were compared directly with those in GeneBank by Blast search (NCBI, http://www.ncbi.nlm.nih.gov/).

### Real-time PCR analysis

On day 21 and 36, all rat fecal samples were collected for real-time PCR analysis. The abundance of specific bacterial groups in the rat fecal samples including *Lactobacillus*, *Bacteroides*, *Enterococcus*, *Enterobacteriaceae*, *Bifidobacterium*, *Clostridium*, *Prevotella* and *Ruminococcus* were measured by real-time PCR detection system (Takara, Japan). The specific primers were showed in S1 Table. Plasmid DNAs were obtained by PCR products cloning with the specific primer sets and used as positive controls. No false-positive results were obtained for the plasmid DNA-free controls, showing that there was no reagent contamination. Each reaction was run in triplicate.

### Fecal microbial transplantation

Ten gram fresh fecal samples from the control group were collected using sterile tubes every morning. Fecal samples were blended with 20 mL sterile warm (37°C) normal saline for 1 minute using a conventional blender. This blended fecal mixture was then centrifuged at 1000 g for 5 min, the supernatant was collected and OD value was tested at 620 nm. This supernatant was prepared for FMT. Twenty four hours after the last dose of letrozole, each rat of the PCOS FMT group was administered a gavage of fecal supernatant with 2×10^9^ fecal microbiota for 14 consecutive days. Meanwhile, each rat of the control group and PCOS group was administered a gavage of normal saline.

### *Lactobacillus* transplantation

*Lactobacillus* were separated from healthy rat’s cecum contents and cultured in facultative anaerobic culture for 48 h, then the bacterial solution was collected and OD value was tested at 620 nm. This bacterial solution was centrifuged at 5000 g for 3 min then the bacteria were collected and resuspended in saline. Each rat of the *Lactobacillus* group was administered a gavage of 2×10^9^ *Lactobacillus* daily for 14 consecutive days.

### Morphological observation

On day 36 (24 h after the last dose of FMT or *Lactobacillus* transplantation), all rats were sacrificed. The ovarian morphological changes were determined as previously described [28]. Ovarian tissue samples were obtained and fixed in 10% neutral-buffered formalin, embedded in paraffin and sectioned at 4 μm. Every 10th section (n = 8) was mounted on a glass slide, stained with hematoxylin/eosinand and analyzed using an Olympus DP73 microscope (Olympus, Tokyo, Japan) by two persons blinded to the origin of sections. Cystic follicles were defined according to criteria proposed previously [29] as those follicles devoid of oocytes, displaying a large antral cavity, an enlarged thecal cell layer, and a thin granulosa cell compartment containing apparently healthy cells.

### Blood sampling for sex steroids

After all rats were sacrificed, Trunk blood samples were obtained and sera were kept at -80°C for subsequent experiments. Estrone, estradiol, testosterone and androstenedione concentrations were determined using enzyme-linked immunosorbent assay (ELISA) kit (Uscn life Science, Wuhan, China). Sensitivities of assays were 9.17 pg/mL, 4.75 pg/mL, 47.2 pg/mL, 46.2 pg/mL for estrone, estradiol, testosterone and androstenedione, respectively. For each hormone, intra-and inter assay coeffients of variation were<10% and<12%, respectively. Each sample was tested in triplicate.

### Statistical analyses

Statistical analyses were performed using SPSS 15.0 software. Data were expressed as Mean ± sem. One-way ANOVA followed by the correction of *p* values with Dunnett’s *post hoc test* was used to determine the significance of data. *P* values<0.05 was considered to be statistically significant.

## Results

### DGGE analysis

**Fig 2:**
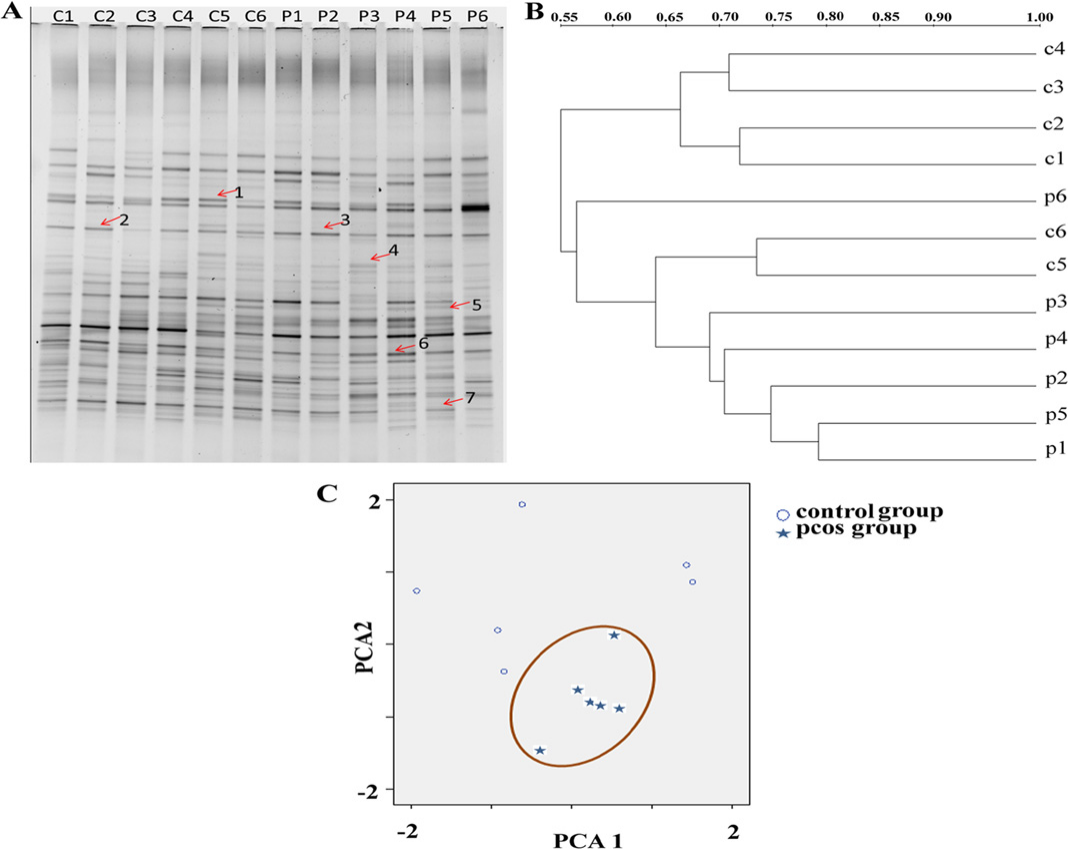
Microbiota profiles in PCOS rats. (A) DGGE profiles in the fecal microbiota of control group and PCOS group (control group: C1-C6; PCOS group: P1-P6). Arrows 1–7 were the bands selected for sequence. (B) Cluster analysis of the DGGE profiles. The dendrogram was constructed using UPGMA method. (C) Principal component analysis (PCA) of fecal microbiota based on DGGE fingerprints. Samples grouped in a solid circle represented fecal microbiota of PCOS group.

To characterize gut microbiota shift in PCOS rats, we performed a global survey of the microbiota in fecal samples. Genetic fingerprints of the gut bacterial communities generated by DGGE analysis showed shifts of the bacterial composition and diversity in feces. It could be observed that some bands became more intense, such as band 3 (Fig 2A). However, the changes of the DGGE band profiles were difficult to be quantified by observation. Therefore, we utilized similarity and Unweighted Pair Group Method with Arithmetic Mean (UPGMA) as a cluster method to demonstrate band pattern similarity. The similarity matrix indicated a 60.1% and 66.9% similarity within the control group and PCOS group, respectively, but only a 58.3% similarity between the two groups (data not shown). Clustering analysis based on the similarity indices showed that PCOS group and the control group clustered in a different branch (Fig 2B). Though two PCOS rats were clustered in the control group, they were in different branches. Principal component analysis (PCA) of DGGE fingerprints further confirmed the differences of gut microbiota between PCOS group and control group. Microbiota structure of the PCOS group showed a separation from the control group by PCA axis 1 and 2. Gut microbiota in PCOS group differed considerably from the control group by moving towards right of PCA axis 1 and down of PCA axis 2 (Fig 2C). These findings indicated that PCOS resulted in a redistribution of the relative abundances of bacterial phyla with the gut microbiota.

### Sequence analysis

Seven bands were selected and cut for sequencing in DGGE gels because the mean integrated optical densities (IOD) of these bands were different between groups. These seven bands were the top seven bands with the greatest density differences between the control and PCOS group. The results were showed in S2 Table. The bands were assigned to bacterial species based on the highest sequence similarity match to GeneBank sequences obtained by BLAST analysis. In order to verify the resolution capability of DGGE, bands in the same position but in different lanes (C2-2 and P2-3) were cut and sequenced. Band 2 and 3 were identified as *Prevotella melaninogenica ATCC 25845* with 93% similarity, indicating that the DGGE gel separated V3 16S rRNA genes from different bacteria effectively. As shown in DGGE profiles, the separated and strong bands in PCOS group were identified as *Pseudomonas monteilii SB3101*, *Roseburia intestinalis XB6B4*, *Prevotella melaninogenica ATCC25845* and *Prevotella denticola F0289*. The weak bands in PCOS group were identified as *Lactobacillus johnsonii NCC533* and *Ruminococcus torques L2-14* (Table 1).

**Table 1:** Sequence identities of PCR amplicons derived from DGGE gels.

Selected bands	Organism with highest sequence homology, sequence accession no. (% homology)	Bacterial Phyla	GeneBank accession number
1	*Lactobacillus johnsonii NCC 533* (94)	Firmicutes	NC_005362
2	*Prevotella melaninogenica ATCC 25845*(93)	Bacteroidetes	NC_014371
3	*Prevotella melaninogenica ATCC 25845*(93)	Bacteroidetes	NC_014371
4	*Pseudomonas monteilii SB3101*(99)	Proteobacteria	NC_023076
5	*Roseburia intestinalis XB6B4*(99)	Firmicutes	NC_021012
6	*Prevotella denticola F0289*(93)	Bacteroidetes	NC_015311
7	*Ruminococcus torques L2-14*(97)	Firmicutes	NC_021015

### Real-time PCR analysis

*Enterococcus*, *Bacteroides*, *Enterobacteriaceae*, *Bifidobacterium* and *Clostridium* genus in fecal samples were chosen for further quantitative analysis on the basis of their important status in the gut; *Lactobacillus*, *Prevotella* and *Ruminococcus* genus were selected because of the results of DGGE analysis and sequence analysis. On day 21, the copy number of *Lactobacillus*, *Ruminococcus* and *Clostridium* were lower and *Prevotella* was higher in PCOS group when compared with the control group. There were no significant differences in the copy number of *Bifidobacterium*, *Escherichia coli*, *Enterococcus* and *Bacteroides* in the two groups (Table 2).

**Table 2:** Quantitation analyses of selected bacterial groups in fecal samples on days 21 and 36 by real-time PCR.

Bacterium	Control group (Mean±sem)	PCOS group (Mean±sem)	Lactobacillus treatment group (Mean±sem)	FMT group (Mean±sem)
*Bifidobacterium* 21d	6.00±0.03	5.99±0.03	6.02±0.01	5.99±0.02
*Bifidobacterium* 36d	6.01±0.10	6.11±0.15	5.93±0.09	5.96±0.06
*Escherichia coli* 21d	4.74±0.07	4.66±0.12	4.99±0.06	4.50±0.09
*Escherichia coli* 36d	4.87±0.34	4.63±0.45	4.55±0.33	4.62±0.43
*Enterococcus* 21d	4.43±0.03	4.18±0.05	4.09±0.04	4.12±0.03
*Enterococcus* 36d	4.52±0.25	4.16±0.39	4.03±0.12	4.03±0.27
*Lactobacillus* 21d	4.59±0.06	3.70±0.04*	3.59±0.05*	3.56±0.06*
*Lactobacillus* 36d	4.61±0.25	3.67±0.19*	4.45±0.14^#^	4.43±0.10^#^
*Bacteroides* 21d	6.90±0.07	7.04±0.05	6.99±0.04	7.11±0.05
*Bacteroides* 36d	7.09±0.07	7.25±0.03	6.87±0.13	6.68±0.06
*Prevotella* 21d	2.47±0.02	5.08±0.11*	4.97±0.13*	4.95±0.15*
*Prevotella* 36d	2.77±0.55	5.49±0.29*	4.66±0.43^#^*	3.91±0.30^#^*
*Ruminococcus* 21d	5.38±0.07	4.34±0.06*	4.25±0.09*	4.37±0.07*
*Ruminococcus* 36d	5.40±0.12	4.41±0.19*	4.66±0.32*	4.53±0.16*
*Clostridium* 21d	5.03±0.08	4.14±0.10*	4.11±0.05*	4.17±0.08*
*Clostridium* 36d	5.16±0.18	4.15±0.21*	5.46±0.33^#^	5.52±0.20^#^

Data were reported as the average estimate of Log_10_ of fecal PCR target genetic amplicon copy numbers present in 1 g of feces.

* p<0.05 results which are significantly different versus control group

# p<0.05 data are significantly different versus PCOS group.

In order to verify the effects of FMT and *Lactobacillus* transplantation on the composition of gut microbiota in PCOS rats, PCOS rats were treated with FMT or *Lactobacillus* and the bacteria described above were examined. As shown in Table 2, on day 36, the copy number of *Lactobacillus* and *Clostridium* were increased in both FMT group and *Lactobacillus* transplantation group, to levels similar to that of the control group. The abundance of *Prevotella* was decreased in both treated groups and the changes in FMT group were more significant than that in *Lactobacillus* transplantation group. Though *Prevotella* in both groups still have significant differences with the control, it decreased significantly when compared with the PCOS group. There were no significant differences in the copy number of *Bifidobacterium*, *Escherichia coli*, *Enterococcus*, *Bacteroides* and *Ruminococcus* in the two treated groups when compared with PCOS group. These data indicated that PCOS resulted in a significant variation of gut microbiota composition. Both FMT and *Lactobacillus* transplantation were beneficial for restorations of normal gut residents in letrozole induced PCOS rats.

### FMT and *Lactobacillus* transplantation improved estrous cycles in letrozole induced PCOS rats

**Fig 3:**
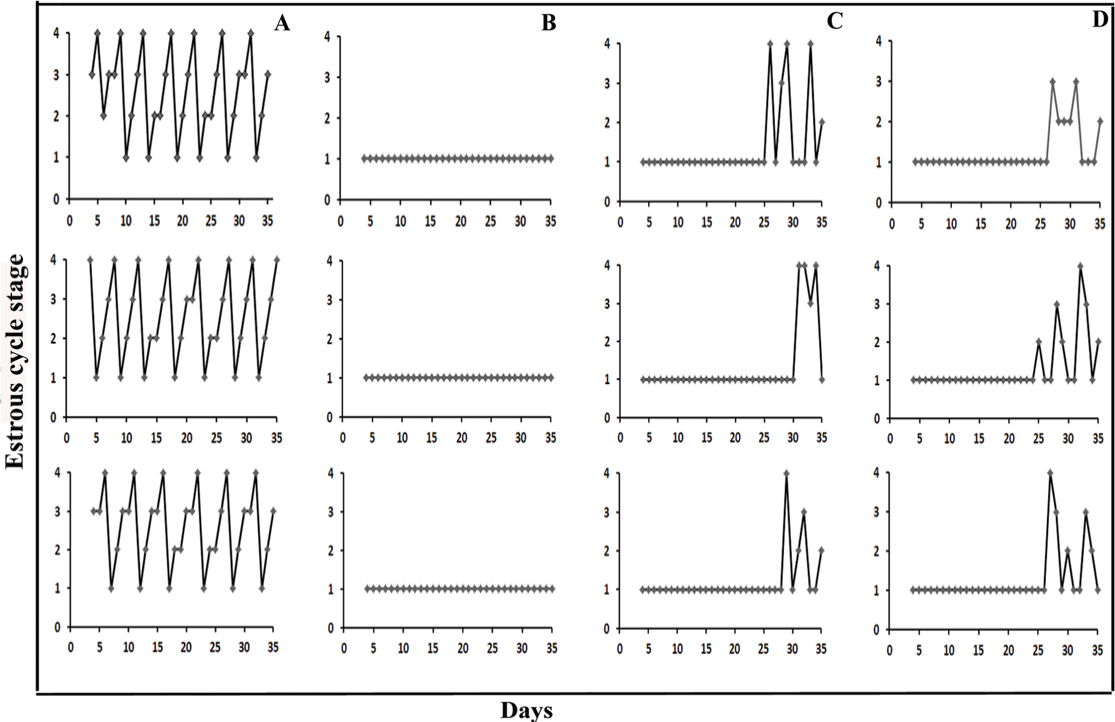
Estrous cycle changes in three representative rats from each group. Cycle stages are as follows: 1, diestrus; 2, proestrus; 3, estrus and 4, metestrus. Groups are as follows: (A) Control group; (B) PCOS group; (C) PCOS *Lactobacillus* transplantation group; (D) PCOS FMT group.

Using daily vaginal smears, estrous cycles were analyzed for all rats. All control rats had regular estrous cycles of 4–5 days, comprising proestrus, estrus, metestrus and diestrus. The PCOS rats were constantly in diestrus stage, exhibiting predominantly leukocytes. On the 7th day after PCOS rats were treated with FMT, epithelial keratinocytes were observed microscopically during estrus in the vaginal smears of 3 rats; on the 14th day epithelial keratinocytes were observed in all of the 8 rats. The estrous cycle changes occurred twice in 5 rats, once in 3 rats. In the PCOS *Lactobacillus* transplantation group, 1 of 8 rats had epithelial keratinocytes on the 7th day; 6 of 8 rats had epithelial keratinocytes on the 14th day; the remaining 2 rats were in diestrus at the time of sacrifice. The estrous cycle changes occurred twice in 2 rats, once in 4 rats, and not at all in 2 rats (Fig 3).

### Ovarian morphological changes

**Fig 4:**
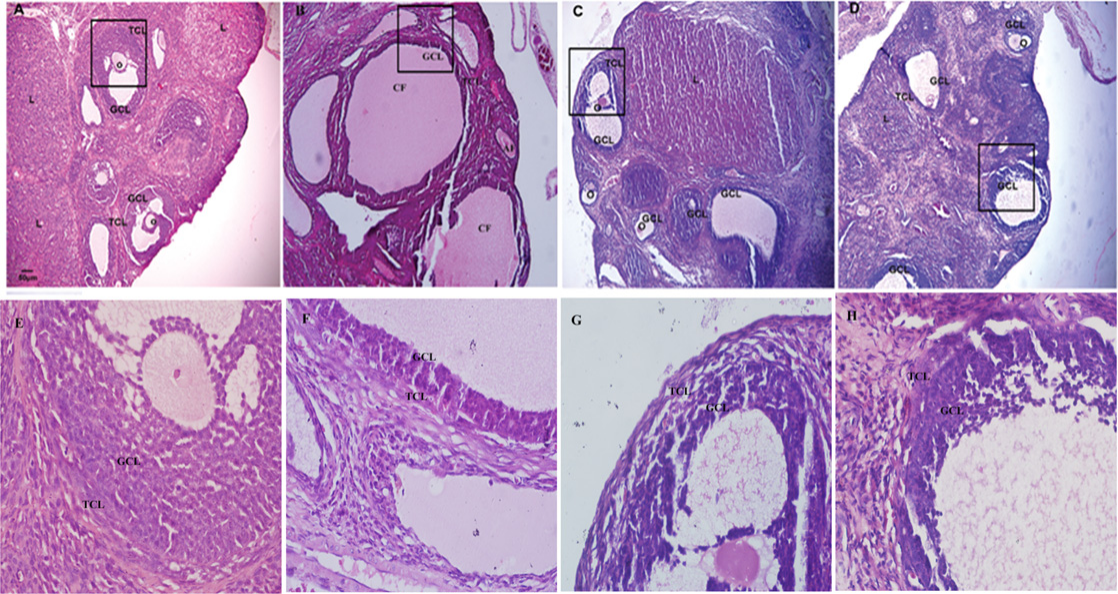
Morphological changes of ovarian tissues. (A) Sections of ovary from the control group had normal appearance. (B) PCOS group showed cystic degenerating follicles with thin granulosa layers. (C) PCOS *Lactobacillus* transplantation group showed increased granulosa layers. (D) PCOS FMT group showed increased granulosa layers and formation of corpora lutea. The larger boxed area in A, B, C, D was shown at higher magnification (400×) in E, F, G, H, respectively. GLC: granular cell layer, TCL: theca cell layer, O: oocyte, L: luteum, AF: atretic follicle. Magnification (100×), scale bar, 50 μm.

Under light microscopy, the control rat ovaries exhibited follicles in various stages of development including secondary follicles, graafian follicles, and fresh corpora lutea. The granulosa within the follicles showed multiple layers. The PCOS rat ovaries showed small follicles in early stage of development and atretic follicles. In addition, many large cysts with virtually no granulosa layer or scant granulosa were observed. The FMT and *Lactobacillus* transplantation rat ovaries showed increased granulosa layers and the formation of corpora lutea (Fig 4 and Table 3).

**Table 3:** Comparison of ovarian morphological changes in experimental and control groups of rats.

	Control group(Mean±sem)	PCOS group(Mean±sem)	*Lactobacillus* treatment group(Mean±sem)	FMT group(Mean±sem)
Cystic follicles (n)	0.50±0.13	7.63±0.24*	4.38±0.22*^#^	3.38±0.20*^#^
Number of corpora lutea (n)	8.50±0.27	0.13±0.06*	4.88±0.23*^#^	6.25±0.22*^#^
Diameter of largest follicle (μm)	115.13±6.32	463.75±33.23*	398.25±23.28*^#^	317.25±22.63*^#^
Thickness of granulosa cell layer (μm)	77.88±2.95	36.63±1.67*	50.50±16.78*^#^	60.87±3.27*^#^

Every 10th section of 8 rats in each group was calculated.

n the mean number for each section in the same group.

*p < 0.05 results which are significantly different versus control group

# p < 0.05 data are significantly different versus PCOS group.

### Steroid concentrations

**Fig 5:**
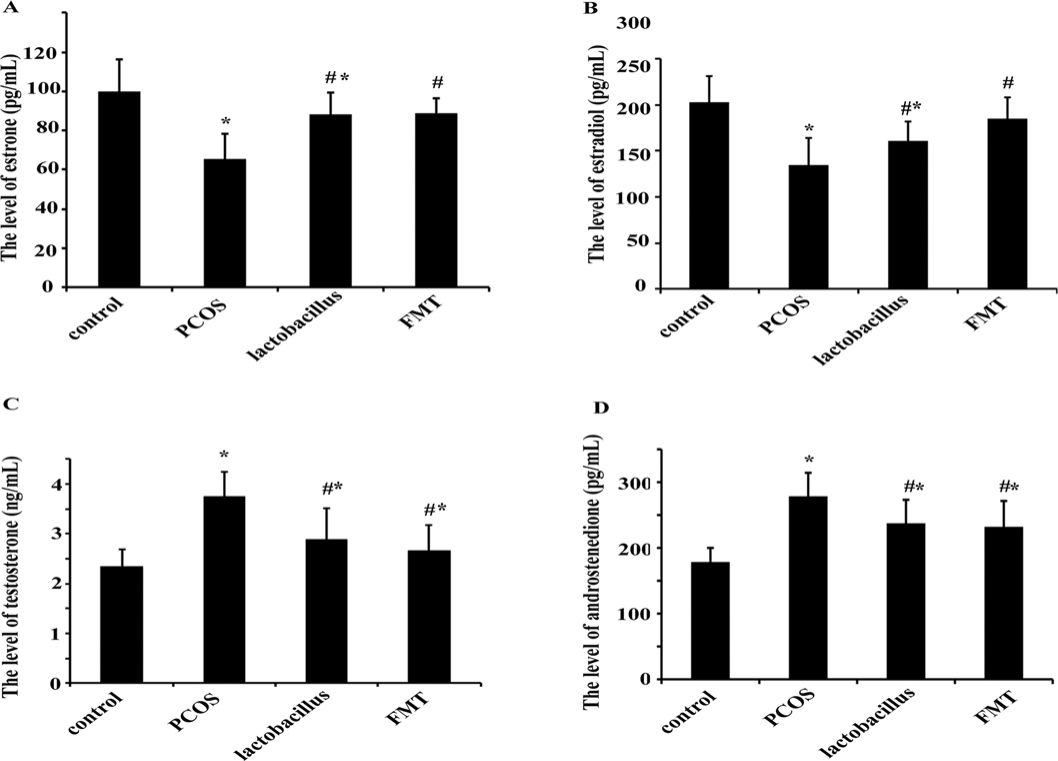
Comparisons of serum sex steroids at 36 days. The concentrations of estrone (A), estradiol (B), androstenedione (C) and testosterone (D) in the serum were quantified with ELISA kit. These data were shown as Mean±sem. * p < 0.*05* versus control group, *# p < 0*.*05* versus PCOS group.

The serum estradiol and estrone concentrations were significantly lower in PCOS rats than those in the control rats. When PCOS rats were treated with FMT or *Lactobacillus*, the estradiol and estrone levels were increased significantly compared with those in PCOS rats (Fig 5A and 5B). FMT was more effective in elevating estradiol and estrone levels than *Lactobacillus* transplantation as there was no significant difference between the FMT group and the control group.

The serum testosterone and androstenedione concentrations were significantly higher in PCOS rats than those in control rats. When PCOS rats were treated with FMT or *Lactobacillus*, the testosterone and androstenedione levels were decreased significantly compared with those in PCOS rats (Fig 5C and 5D). Also FMT was more effective in reducing testosterone and androstenedione levels when compared with *Lactobacillus* transplantation. However, the differences were still statistically significant when compared with the control.

## Discussion

The increasing knowledge of the role of microbiota in sex and gender difference has presented new horizons on the critical role of the enteric microbiota in regulating sex hormones in health and disease state. It has been reported that sex differences in the gut microbiome drived hormone-dependent regulating autoimmunity. Early life exposures determined the sex hormone level and modified progression to autoimmunity in type 1 diabetes mouse (T1D) model. Transplantation of the male microbiome to immature females increased the level of testosterone and formed a testosterone-dependent metabolite profile in the female recipients and robust T1D protection [18]. Besides the communication between gut microbiota and testosterone occurring in immune system, gut microbiota has a direct effect over endocrine system. Clarke. *et al* found that male germ free mice, unlike females, exhibited a significant high level of 5-hydroxytryptamine and its metabolite in hippocampal compared with conventionally housed control animals [28]. Collectively, these studies indicated the bi-direction regulation of gut microbiota and endocrine systems.

Excess androgen production and relatively insufficient estradiol are major traits for PCOS patients and essential for follicle development [29]. Such hormone changes in PCOS are likely associated with dysbiosis of gut microbiota. In this study, letrozole-induced PCOS rat model was used to evaluate the gut microbiota and sex hormone concentrations. Letrozole is a nonsteroidal aromatase inhibitor that reduces conversion of androgens to estrogens in the ovary, resulting in increased testosterone and decreased estrone production [30]. Decreased aromatase activity in the ovary is one of the pathophysiologic hypotheses of PCOS development [31, 32]. In letrozole-induced PCOS rats, the serum estradiol and progesterone levels were reduced, testosterone and luteinizing hormone (LH) levels were elevated in a dose-dependent manner [26]. Despite the fact that letrozole rats had a similar metabolic characteristic to the controls, the endocrine hormone profile of this animal model in several ways was similar to human PCOS. Recently, letrozole-induced PCOS rat model was used in several papers to evaluate effects of new therapeutic methods for PCOS [33, 34], which illustrated the recognition of this animal model from another angle. Therefore in this study, we utilized this animal model to study the relationship between gut microbiota and PCOS.

The present study demonstrated that the microbiota composition of letrozole-induced PCOS rats and control rats were divided into different clusters, and PCOS group displayed a relatively high homology indicating common characteristics of PCOS. Sequence analysis of the excised bands showed that the strong bands in PCOS group were related to *Pseudomonas*, *Roseburia* and *Prevotella*, the weak bands in PCOS group were related to *Lactobacillus* and *Ruminococcus*. DGGE analysis is a semiquantitative technique, the band density does not accurately relate to its abundance. Therefore, real-time PCR was utilized to obtain a quantitative estimation of *Lactobacillus*, *Enterococcus*, *Bacteroides*, *Enterobacteriaceae*, *Bifidobacterium*, *Clostridium*, *Prevotella* and *Ruminococcus* genus. The results revealed that *Lactobacillus*, *Ruminococcus* and *Clostridium* were lower and *Prevotella* was higher in PCOS group, there were no statistically significant differences for *Bifidobacterium*, *Escherichia coli*, *Enterococcus* and *Bacteroides* in PCOS groups when compared with the control group.

*Prevotella* species are constituents of host-associated ecosystems in the respiratory tract, oral cavity and genital tract. These organisms contributed to polymicrobial infections such as chronic sinusitis, periodontitis and bacterial vaginosis [35–37]. Recent investigations have revealed that males not females have greater odds of carrying *Prevotella intermedia* in oral cavity, suggesting an association between sex hormone and carriage of *Prevotella* [38]. In women with PCOS, *Prevotella intermedia* serum antibody levels were higher than healthy women [39]. It has been hypothesized that testosterone metabolites contributed to *Prevotella* growth and virulence [40]. However, there is no evidence to support this hypothesis. *Prevotella intermedia* were capable of stimulating 5α-reductase activity in human gingival fibroblasts, leading to the synthesis of dihydrotestosterone form testosterone and androstenedione [41, 42]. In this study, with the testosterone and androstenedione levels increased in PCOS group, the copy number of *Prevotella* also increased, which is consistent with previous study. To further understand effects of microbiota interventions on PCOS, PCOS rats were treated with FMT and *Lactobacillus* transplantation. The results showed that with the copy number of *Prevotella* decrease the testosterone and androstenedione levels also decreased. The relationship between *Prevotella* and androgen level needs further study in letrozole-induced PCOS rat model.

*Lactobacillus* plays an important role in the maintenance of human health by stimulating the natural immunity and contributing to the balance of microbiota [43]. A previous study showed that postmenopausal women with a more diverse gut microbiome exhibited elevated urinary estrogens and estrogen metabolites [44]. In the present study, with the decrease of estradiol and estrone levels in PCOS rats, the colonization of *Lactobacillus* in the gut was decreased. When PCOS rats were treated with FMT and *Lactobacillus* transplantation, with the increase of *Lactobacilli*, estradiol and estrone levels were increased significantly. There is a limitation of our study: ELISA sensitivity can fail to detect low levels of sex steroids and could not analyze the serum steroids in female rodents according to estrus cycle stage [45]. However, the available data indicated that sex steroids concentrations could be regulated by microbiota interventions in PCOS rat model.

Gut microbiota such as *Bifidobacteria* and *Lactobacillus* are often referred to as “good bacteria” as they exert health-promoting properties. It is proposed that increase of *Lactobacillus* in the gut lead to production of short chain fatty acids that improve gut health, increasing the barrier function of the gut and reducing translocation of bacterial endotoxins across the gut wall where they could produce inflammation and insulin resistance [19]. Probiotic supplementation *(Lactobacillus casei Shirota (LcS)*) could prevent high-fat, over-feeding induced insulin resistance in human subjects [46]. Considering the beneficial effect of *Lactobacillus* on insulin resistance and the disorder is associated with PCOS, the present study used *Lactobacillus* transplantation and FMT to treat PCOS rats. The results showed that after treating PCOS rats with *Lactobacillus* transplantation and FMT, the serum androgens reduced, estrous cycles improved and the ovarian functions normalized. These data suggested that FMT and *Lactobacillus* transplantation were helpful for the treatments of PCOS rats.

In conclusion, we observed the gut microbiota shift in letrozole induced PCOS rat model and found that dysbiosis of gut microbiota was associated with sex hormone levels, estrus cycles and ovarian morphological changes. Furthermore, microbiota interventions by FMT and *Lactobacillus* transplantation could decrease the androgen level and increase the estrogen level in blood serum, improve ovarian disorder and estrus cycles in PCOS rats. In addition, compared with *Lactobacillus* transplantation group, FMT group had better recovery effects on sex hormone levels and estrus cycles. This may be caused by the unknown microbiota in the feces. We hope that this study could raise concerns about the important roles of gut microbiota in the etiology and therapeutic effects in PCOS.

## Supporting Information

S1 Table 16S rRNA gene-targeted group-specific primers used in this study. (DOCX)

S2 Table Quantity analysis of the sequenced bands. a IOD: Integrated optical density; b Calculated as control IOD/PCOS IOD.
(DOCX)

## References

[1] Norman RJ, Dewailly D, Legro RS, Hickey TE. Polycystic ovary syndrome. Lancet. 2007;370(9588):685–97. 10.1016/S0140-6736(07)61345-2 . 17720020

[2] Homburg R. Polycystic ovary syndrome. Best Pract Res Clin Obstet Gynaecol. 2008;22(2):261–74. 10.1016/j.bpobgyn.2007.07.009 . 17804299

[3] Dokras A. Cardiovascular disease risk in women with PCOS. Steroids. 2013;78(8):773–6. 10.1016/j.steroids.2013.04.009 . 23624351

[4] Hsu MI. Changes in the PCOS phenotype with age. Steroids. 2013;78(8):761–6. 10.1016/j.steroids.2013.04.005 . 23624031

[5] Lim SS, Norman RJ, Davies MJ, Moran LJ. The effect of obesity on polycystic ovary syndrome: a systematic review and meta-analysis. Obes Rev. 2013;14(2):95–109. 10.1111/j.1467-789X.2012.01053.x . 23114091

[6] Balen A. The pathophysiology of polycystic ovary syndrome: trying to understand PCOS and its endocrinology. Best Pract Res Clin Obstet Gynaecol. 2004;18(5):685–706. 10.1016/j.bpobgyn.2004.05.004 . 15380141

[7] Ehrmann DA. Polycystic ovary syndrome. N Engl J Med. 2005;352(12):1223–36. 10.1056/NEJMra041536 . 15788499

[8] Goodarzi MO, Dumesic DA, Chazenbalk G, Azziz R. Polycystic ovary syndrome: etiology, pathogenesis and diagnosis. Nat Rev Endocrinol. 2011;7(4):219–31. 10.1038/nrendo.2010.217 . 21263450

[9] Franks S, Gharani N, Waterworth D, Batty S, White D, Williamson R, et al The genetic basis of polycystic ovary syndrome. Hum Reprod. 1997;12(12):2641–8. . 9455828 10.1093/humrep/12.12.2641

[10] Fox R, Ryan A. Polycystic ovary syndrome: not ovarian, not simple, unkind. Hum Fertil (Camb). 2002;5(1 Suppl):S28–32. . 11897912 10.1080/1464727022000199881

[11] Haag LM, Fischer A, Otto B, Plickert R, Kühl AA, Göbel UB, et al Intestinal microbiota shifts towards elevated commensal Escherichia coli loads abrogate colonization resistance against Campylobacter jejuni in mice. PLOS One. 2012;7(5):e35988 10.1371/journal.pone.0035988 22563475 PMC3341396

[12] McNeil NI. The contribution of the large intestine to energy supplies in man. Am J Clin Nutr. 1984;39(2):338–42. . 6320630 10.1093/ajcn/39.2.338

[13] Maloy KJ, Powrie F. Intestinal homeostasis and its breakdown in inflammatory bowel disease. Nature. 2011;474(7351):298–306. 10.1038/nature10208 . 21677746

[14] Wang Z, Klipfell E, Bennett BJ, Koeth R, Levison BS, Dugar B, et al Gut flora metabolism of phosphatidylcholine promotes cardiovascular disease. Nature. 472(7341):57–63. Epub 2011/04/09. doi: nature09922 [pii] 10.1038/nature09922 21475195 PMC3086762

[15] Markle JG, Frank DN, Mortin-Toth S, Robertson CE, Feazel LM, Rolle-Kampczyk U, et al Sex differences in the gut microbiome drive hormone-dependent regulation of autoimmunity. Science. 339(6123):1084–8. Epub 2013/01/19. doi: science.1233521 [pii] 10.1126/science.1233521 . 23328391

[16] Moran CP, Shanahan F. Gut microbiota and obesity: role in aetiology and potential therapeutic target. Best Pract Res Clin Gastroenterol. 28(4):585–97. Epub 2014/09/10. doi: S1521-6918(14)00083-3 [pii] 10.1016/j.bpg.2014.07.005 . 25194177

[17] Flak MB, Neves JF, Blumberg RS. Immunology. Welcome to the microgenderome. Science. 2013;339(6123):1044–5. 10.1126/science.1236226 23449586 PMC4005781

[18] Markle JG, Frank DN, Mortin-Toth S, Robertson CE, Feazel LM, Rolle-Kampczyk U, et al Sex differences in the gut microbiome drive hormone-dependent regulation of autoimmunity. Science. 2013;339(6123):1084–8. 10.1126/science.1233521 . 23328391

[19] Tremellen K, Pearce K. Dysbiosis of Gut Microbiota (DOGMA)—a novel theory for the development of Polycystic Ovarian Syndrome. Med Hypotheses. 2012;79(1):104–12. 10.1016/j.mehy.2012.04.016 . 22543078

[20] Yang S, Li W, Challis JR, Reid G, Kim SO, Bocking AD. Probiotic Lactobacillus rhamnosus GR-1 supernatant prevents lipopolysaccharide-induced preterm birth and reduces inflammation in pregnant CD-1 mice. Am J Obstet Gynecol. 211(1):44 e1–e12. Epub 2014/02/04. doi: S0002-9378(14)00058-1 [pii] 10.1016/j.ajog.2014.01.029 . 24486224

[21] Andreasen AS, Larsen N, Pedersen-Skovsgaard T, Berg RM, Moller K, Svendsen KD, et al Effects of Lactobacillus acidophilus NCFM on insulin sensitivity and the systemic inflammatory response in human subjects. Br J Nutr. 104(12):1831–8. Epub 2010/09/08. doi: S0007114510002874 [pii] 10.1017/S0007114510002874 . 20815975

[22] Johnson-Henry KC, Donato KA, Shen-Tu G, Gordanpour M, Sherman PM. Lactobacillus rhamnosus strain GG prevents enterohemorrhagic Escherichia coli O157:H7-induced changes in epithelial barrier function. Infect Immun. 2008;76(4):1340–8. Epub 2008/01/30. doi: IAI.00778-07 [pii] 10.1128/IAI.00778-07 18227169 PMC2292865

[23] Parassol N, Freitas M, Thoreux K, Dalmasso G, Bourdet-Sicard R, Rampal P. Lactobacillus casei DN-114 001 inhibits the increase in paracellular permeability of enteropathogenic Escherichia coli-infected T84 cells. Res Microbiol. 2005;156(2):256–62. Epub 2005/03/08. doi: S0923-2508(04)00263-3 [pii] 10.1016/j.resmic.2004.09.013 . 15748992

[24] Vrieze A, Van Nood E, Holleman F, Salojarvi J, Kootte RS, Bartelsman JF, et al Transfer of intestinal microbiota from lean donors increases insulin sensitivity in individuals with metabolic syndrome. Gastroenterology. 143(4):913–6 e7. Epub 2012/06/26. doi: S0016-5085(12)00892-X [pii] 10.1053/j.gastro.2012.06.031 . 22728514

[25] Li M, Liang P, Li Z, Wang Y, Zhang G, Gao H, et al Fecal microbiota transplantation and bacterial consortium transplantation have comparable effects on the re-establishment of mucosal barrier function in mice with intestinal dysbiosis. Front Microbiol. 6:692 Epub 2015/07/29. 10.3389/fmicb.2015.00692 26217323 PMC4493656

[26] Kafali H, Iriadam M, Ozardali I, Demir N. Letrozole-induced polycystic ovaries in the rat: a new model for cystic ovarian disease. Arch Med Res. 2004;35(2):103–8. 10.1016/j.arcmed.2003.10.005 . 15010188

[27] Joossens M, Huys G, Cnockaert M, De Preter V, Verbeke K, Rutgeerts P, et al Dysbiosis of the faecal microbiota in patients with Crohn's disease and their unaffected relatives. Gut. 2011;60(5):631–7. 10.1136/gut.2010.223263 . 21209126

[28] Clarke G, Grenham S, Scully P, Fitzgerald P, Moloney RD, Shanahan F, et al The microbiome-gut-brain axis during early life regulates the hippocampal serotonergic system in a sex-dependent manner. Mol Psychiatry. 2013;18(6):666–73. 10.1038/mp.2012.77 . 22688187

[29] Lebbe M, Woodruff TK. Involvement of androgens in ovarian health and disease. Mol Hum Reprod. 2013;19(12):828–37. 10.1093/molehr/gat065 24026057 PMC3843026

[30] Corbin CJ, Trant JM, Walters KW, Conley AJ. Changes in testosterone metabolism associated with the evolution of placental and gonadal isozymes of porcine aromatase cytochrome P450. Endocrinology. 1999;140(11):5202–10. 10.1210/endo.140.11.7140 . 10537150

[31] Chen J, Shen S, Tan Y, Xia D, Xia Y, Cao Y, et al The correlation of aromatase activity and obesity in women with or without polycystic ovary syndrome. J Ovarian Res. 2015;8(1):11 10.1186/s13048-015-0139-1 25881575 PMC4392749

[32] Diamanti-Kandarakis E. Polycystic ovarian syndrome: pathophysiology, molecular aspects and clinical implications. Expert Rev Mol Med. 2008;10:e3 10.1017/S1462399408000598 . 18230193

[33] Sun J, Jin C, Wu H, Zhao J, Cui Y, Liu H, et al Effects of electro-acupuncture on ovarian P450arom, P450c17α and mRNA expression induced by letrozole in PCOS rats. PLOS One. 2013;8(11):e79382 10.1371/journal.pone.0079382 24260211 PMC3832614

[34] Küpeli Akkol E, İlhan M, Ayşe Demirel M, Keleş H, Tümen I, Süntar İ. Thuja occidentalis L. and its active compound, α-thujone: Promising effects in the treatment of polycystic ovary syndrome without inducing osteoporosis. J Ethnopharmacol. 2015 10.1016/j.jep.2015.03.029 . 25818694

[35] Brook I. Microbiology and antimicrobial management of sinusitis. J Laryngol Otol. 2005;119(4):251–8. 10.1258/0022215054020304 . 15949076

[36] Oakley BB, Fiedler TL, Marrazzo JM, Fredricks DN. Diversity of human vaginal bacterial communities and associations with clinically defined bacterial vaginosis. Appl Environ Microbiol. 2008;74(15):4898–909. 10.1128/AEM.02884-07 18487399 PMC2519371

[37] Zambon JJ, Reynolds HS, Slots J. Black-pigmented Bacteroides spp. in the human oral cavity. Infect Immun. 1981;32(1):198–203. 6111541 10.1128/iai.32.1.198-203.1981 PMC350607

[38] Umeda M, Chen C, Bakker I, Contreras A, Morrison JL, Slots J. Risk indicators for harboring periodontal pathogens. J Periodontol. 1998;69(10):1111–8. 10.1902/jop.1998.69.10.1111 . 9802709

[39] Akcalı A, Bostanci N, Özçaka Ö, Öztürk-Ceyhan B, Gümüş P, Buduneli N, et al Association between polycystic ovary syndrome, oral microbiota and systemic antibody responses. PLOS One. 2014;9(9):e108074 10.1371/journal.pone.0108074 25232962 PMC4169459

[40] Kumar PS. Sex and the subgingival microbiome: do female sex steroids affect periodontal bacteria? Periodontol 2000. 2013;61(1):103–24. 10.1111/j.1600-0757.2011.00398.x . 23240946

[41] Soory M, Ahmad S. 5 alpha reductase activity in human gingiva and gingival fibroblasts in response to bacterial culture supernatants, using [14C]4-androstenedione as substrate. Arch Oral Biol. 1997;42(4):255–62. . 9222443 10.1016/s0003-9969(97)00028-9

[42] Soory M. Bacterial steroidogenesis by periodontal pathogens and the effect of bacterial enzymes on steroid conversions by human gingival fibroblasts in culture. J Periodontal Res. 1995;30(2):124–31. . 7776153 10.1111/j.1600-0765.1995.tb01261.x

[43] McFarland LV. Beneficial microbes: health or hazard? Eur J Gastroenterol Hepatol. 2000;12(10):1069–71. . 11057450 10.1097/00042737-200012100-00001

[44] Fuhrman BJ, Feigelson HS, Flores R, Gail MH, Xu X, Ravel J, et al Associations of the fecal microbiome with urinary estrogens and estrogen metabolites in postmenopausal women. J Clin Endocrinol Metab. 99(12):4632–40. Epub 2014/09/12. 10.1210/jc.2014-2222 25211668 PMC4255131

[45] Nilsson ME, Vandenput L, Tivesten A, Norlen AK, Lagerquist MK, Windahl SH, et al Measurement of a Comprehensive Sex Steroid Profile in Rodent Serum by High-Sensitive Gas Chromatography-Tandem Mass Spectrometry. Endocrinology. 156(7):2492–502. Epub 2015/04/10. 10.1210/en.2014-1890 . 25856427

[46] Hulston CJ, Churnside AA, Venables MC. Probiotic supplementation prevents high-fat, overfeeding-induced insulin resistance in human subjects. Br J Nutr. 2015;113(4):596–602. 10.1017/S0007114514004097 25630516 PMC4339038

