# Supplementary material for: Association between Polycystic Ovary Syndrome and Gut Microbiota
Source: PLoS One. 2016 Apr 19;11(4):e0153196. doi: 10.1371/journal.pone.0153196 (PMC4836746; doi:10.1371/journal.pone.0153196)
Supplement: S1 Table — (DOCX) [file pone.0153196.s001.docx]

**Supplement Table1. 16S rRNA gene-targeted group-specific primers used in this study**

The bacterial strains used as positive and negative controls in

this study included Atopobiumparvulum

The bacterial strains used as positive and negative controls in

this study included Atopobiumparvulum

The bacterial strains used as positive and negative controls in

this study included Atopobiumparvulum

| Target bacterial group | Primer | Sequence  (5’-3’) | References |
| --- | --- | --- | --- |
| *Bifidobacterium* | Bifid-F  Bifid-R | TCGCGTCYGGTGTGAAAG  GGTGTTCTTCCCGATATCTACA | [1]  [2]  [3]  [4]  [1]  [5]  [6]  [7] |
| *Escherichia coli* | Ecoli-F  Ecoli-R | CATGCCGCGTGTATGAAGAA  CGGGTAACGTCAATGAGCAAA |  |
| *Enterococcus* | Entero-F  Entero-R | AGAAATTCCAAACGAACTTG  CAGTGCTCTACCTCCATCATT |  |
| *Lactobacillus* | Lacto-F  Lacto-R | AGCAGTAGGGAATCTTCCA  CACCGCTACACATGGAG |  |
| *Bacteroides* | Bact-F  Bact-R | GGTGTCGGCTTAAGTGCCAT  CGGA(C/T)GTAAGGGCCGTGC |  |
| *Prevotella* | Prevo-F  Prevo-R | CACRGTAAACGATGGATGCC  GGTCGGGTTGCAGACC |  |
| *Ruminococcus* | Rumino-F  Rumino-R | GGACGATAATGACGGTACTT  GCAATCYGAACTGGGACAAT |  |
| *Clostridium* | Clostri-F  Clostri-R | GCACAAGCAGTGGAGT  CTTCCTCCGTTTTGTCAA |  |

**References**

1.Rinttila T, Kassinen A, Malinen E, Krogius L, Palva A. Development of an extensive set of 16S

rDNA-targeted primers for quantification of pathogenic and indigenous bacteria in faecal samples

by real-time PCR. J Appl Microbiol 2004;97(6): 1166-1177.

2.Huijsdens XW, Linskens RK, Mak M, Meuwissen SG, Vandenbroucke-Grauls CM, [Savelkoul PH](http://www.ncbi.nlm.nih.gov/pubmed/?term=Savelkoul%20PH%5BAuthor%5D&cauthor=true&cauthor_uid=12454130).

Quantification of bacteria adherent to gastrointestinal mucosa by real-time PCR. J Clin Microbiol

2002 Dec;40(12): 4423-4427.

3. Ludwig W, Schleifer KH. How quantitative is quantitative PCR with respect to cell counts? Syst Appl

Microbiol 2000 Dec;23(4): 556-562.

4. Walter J, Hertel C, Tannock GW, Lis CM, Munro K, Hammes WP. Detection of Lactobacillus,

Pediococcus, Leuconostoc, and Weissella species in human feces by using group-specific PCR

primers and denaturing gradient gel electrophoresis. Appl Environ Microbiol2001 Jun;67(6):

2578-2585.

5. Matsuki T, Watanabe K, Fujimoto J, Miyamoto Y, Takada T, Matsumoto K, et al. Development of 16S

rRNA-gene-targeted group-specific primers for the detection and identification of predominant

bacteria in human feces. Appl Environ Microbiol2002 Nov;68(11): 5445-5451.

6. Tajima K, Aminov RI, Nagamine T, Matsui H, Nakamura M, Benno Y. Diet-dependent shifts in the

bacterial population of the rumen revealed with real-time PCR. Appl Environ Microbiol 2001

Jun;67(6): 2766-2774.

7. Zhou L, Li X, Ahmed A, Wu D, Liu L, Qiu J, et al. Gut microbe analysis between hyperthyroid and

healthy individuals. Curr Microbiol 2014 Nov;69(5): 675-680.
