# Supplementary material for: Association between Polycystic Ovary Syndrome and Gut Microbiota
Source: PLoS One. 2016 Apr 19;11(4):e0153196. doi: 10.1371/journal.pone.0153196 (PMC4836746; doi:10.1371/journal.pone.0153196)
Supplement: S2 Table — a IOD: Integrated optical density; b Calculated as control IOD/PCOS IOD. (DOCX) [file pone.0153196.s002.docx]

**Supplement Table 2.** **Quantity analysis of the sequenced bands**

| **Sequenced bands** | **Control**  **IOD^a^** | **PCOS**  **IOD^a^** | **Fold change^b^** |
| --- | --- | --- | --- |
| Band 1 | 2109 | 839 | 2.513707 |
| Band 2,3 | 761 | 1593 | 0.477715 |
| Band 4 | 691 | 1405 | 0.491815 |
| Band 5 | 306 | 672 | 0.455357 |
| Band 6 | 693 | 1711 | 0.405026 |
| Band 7 | 666 | 325 | 2.049231 |
